# Adenocarcinoma originating from long-segment Barrett's esophagus over 15 cm: a series of 3 cases

**DOI:** 10.1186/s40792-020-00995-7

**Published:** 2020-09-29

**Authors:** Naoki Kuwayama, Isamu Hoshino, Hisashi Gunji, Toru Tonooka, Hiroaki Soda, Ryotaro Eto, Nobuhiro Takiguchi, Yoshihiro Nabeya

**Affiliations:** Division of Gastroenterological Surgery, Chiba Cancer Center, 666-2 Nitona-cho, Chuo-ku, Chiba, 260-8717 Japan

**Keywords:** Barrett's esophagus, Adenocarcinoma, Surveillance

## Abstract

**Background:**

Barrett's esophagus (BE) is characterized by presence of columnar epithelium in the lower esophageal mucosa, which originally comprises stratified squamous epithelium. Gastroesophageal reflux disease causes BE and BE adenocarcinoma (BEAC); further, the incidence of BEAC is increasing, especially in developed countries. Long-segment BE (LSBE) has a particularly high carcinogenic potential and necessitates treatment, surveillance, and prevention.

**Case presentation:**

Herein, we report three cases of BEAC originating from LSBE larger than 15 cm. All three patients underwent surgery for the diagnosis of BEAC. A 66-year-old man with advanced esophageal cancer underwent neoadjuvant chemotherapy and subsequent subtotal esophagectomy. The postoperative pathological diagnosis was of poorly differentiated adenocarcinoma with lymph node metastasis (pT3 pN3 pM0 pStage III based on the Union for International Cancer Control TNM Classification 8th edition). Two years after the operation, the patient was diagnosed with recurrence around the celiac artery and underwent chemotherapy. An 83-year-old woman with advanced esophageal cancer underwent subtotal esophagectomy. The postoperative pathological diagnosis was of well-differentiated adenocarcinoma with supraclavicular lymph node metastasis (pT3 pN3 pM1 pStage IV). Two months after the operation, the patient was diagnosed with recurrence in the neck lymph nodes and underwent chemotherapy; however, she died. A 66-year-old man with early-stage esophageal cancer underwent subtotal esophagectomy. A superficial early cancerous lesion was seen over BE. The postoperative pathological diagnosis was of well-differentiated adenocarcinoma without lymph node metastasis (pT1a pN0 pM0 pStage 0). The patient was found to be alive and recurrence-free 3 months after the operation.

**Conclusions:**

BEAC might show good prognosis if detected and treated early. Extremely LSBE is associated with a high incidence of BEAC; therefore, early detection and treatment with close surveillance is essential.

## Background

Barrett's esophagus (BE) is characterized by presence of columnar epithelium in the lower esophageal mucosa, which originally comprises stratified squamous epithelium. Importantly, long-segment Barrett’s esophagus (LSBE) has a particularly high carcinogenic potential. Here, we report three cases of Barrett's esophageal adenocarcinoma (BEAC) originating from LSBE larger than 15 cm.

## Case presentation

### Case 1

**Fig. 1:**
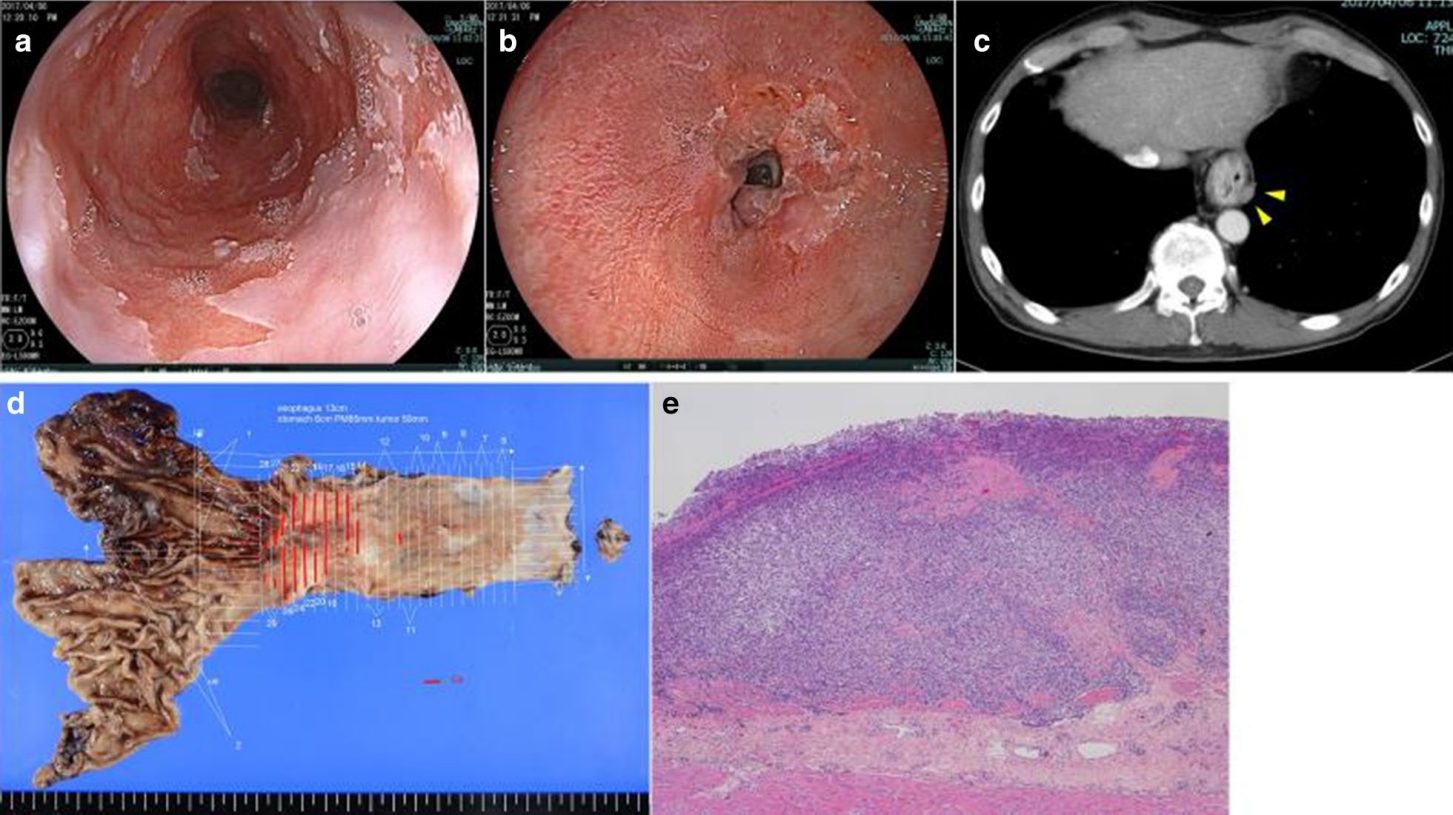
Endoscopy, computed tomography, and histopathological findings for case 1. **a**, **b** Endoscopy revealed a squamocolumnar junction 24 cm from the incisor teeth and a type-3 circumferential tumor on the lower esophagus. **c** Computed tomography detected an enlarged lymph node (#110) but no other distant metastatic sites. **d**, **e** The postoperative pathological diagnosis was of poorly differentiated adenocarcinoma (T3 70 × 45 mm) with lymph node metastasis (pT3 pN3 pM0 pStage III)

A 66-year-old man with esophageal obstruction was admitted to a different hospital. He was referred to our institution following diagnosis of BEAC. Endoscopy revealed a squamocolumnar junction 24 cm from the incisor teeth and a type-3 circumferential tumor on the lower esophagus (Fig. 1). Mucosal biopsy revealed adenocarcinoma.

Computed tomography detected an enlarged lymph node but no other distant metastatic sites. Following diagnosis of cT3N1M0, subtotal esophagectomy with lymph node dissection was performed after three courses of S-1 + oxaliplatin therapy.

The postoperative pathological diagnosis was of poorly differentiated adenocarcinoma (T3 70 × 45 mm) with lymph node metastasis (N3) (pT3 pN3 pM0 pStage III; Fig. 1). Two years after the operation, the patient showed recurrence in the region of the celiac artery and is currently undergoing chemotherapy.

### Case 2

An 83-year-old woman who had undergone upper gastrointestinal endoscopy for detailed examination of anemia was referred to our hospital with a diagnosis of BEAC.

**Fig. 2:**
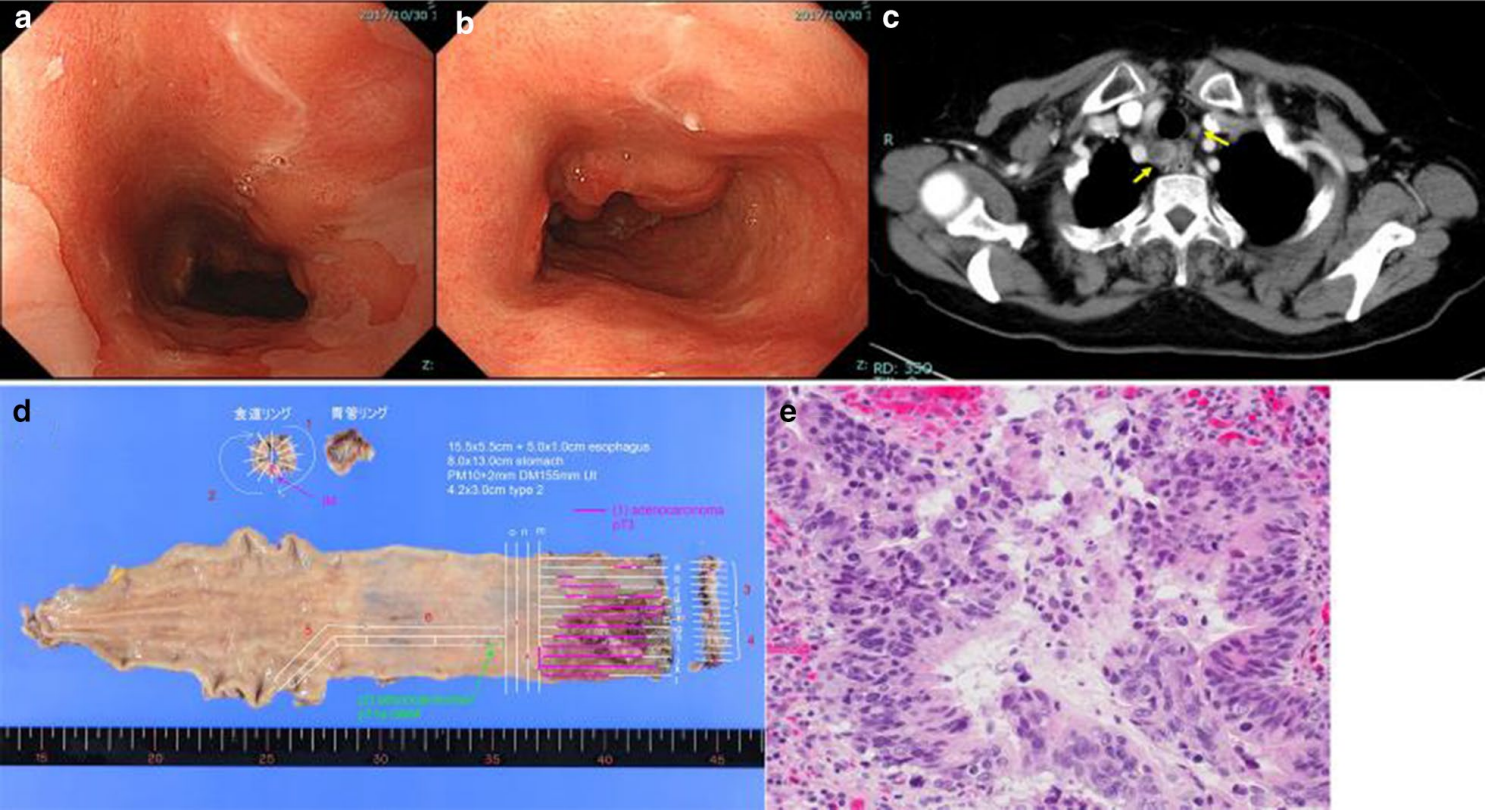
Endoscopy, computed tomography, and histopathological findings for case 2. **a**, **b** Endoscopy revealed a squamocolumnar junction 15 cm from the incisor teeth and a type-2 semicircular tumor on the upper esophagus. **c** Computed tomography detected enlarged cervical paraesophageal lymph nodes (#101) with no other distant metastatic sites. **d**, **e** The postoperative pathological diagnosis was of well-differentiated adenocarcinoma (60 × 52 mm) with supraclavicular lymph nodes metastasis (pT3 pN3 pM1 pStage IV)

Endoscopy revealed a squamocolumnar junction 15 cm from the incisor teeth and a type-2 semicircular tumor on the upper esophagus (Fig. 2). Mucosal biopsy showed adenocarcinoma.

Computed tomography detected enlarged cervical paraesophageal lymph nodes (#101) with no other distant metastatic sites. Following diagnosis of cT3N1M0, subtotal esophagectomy with lymph node and neck dissection were performed. The postoperative pathological diagnosis was of well-differentiated adenocarcinoma (60 × 52 mm) with supraclavicular lymph node metastasis (pT3 pN3 pM1 pStage IV; Fig. 2). Two months after the operation, the patient was diagnosed with recurrence in the neck lymph nodes, for which she underwent chemotherapy; however, she died.

### Case 3

A 66-year-old man diagnosed with BEAC in a periodic medical check‐up was referred to our institution.

**Fig. 3:**
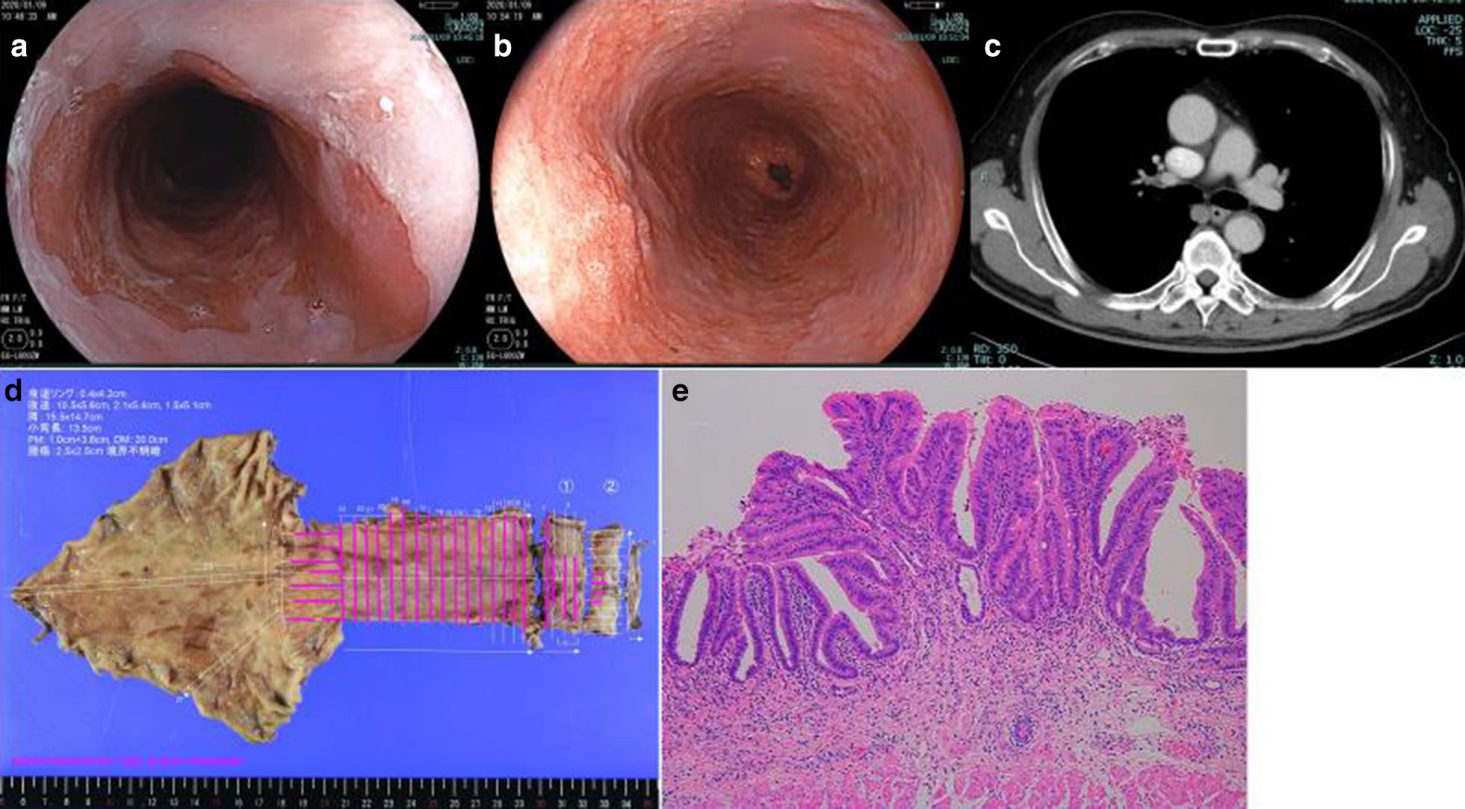
Endoscopy, computed tomography, and histopathological findings for case 3. **a**, **b** Endoscopy revealed a squamocolumnar junction 20 cm from the incisor teeth and a type 0-IIb + IIc tumor with indistinct boundaries 30 cm from the incisor teeth. **c** Computed tomography detected no enlarged lymph nodes and no other distant metastatic sites. **d**, **e** The postoperative pathological diagnosis was of well-differentiated adenocarcinoma (143 × 56 mm) with no lymph node metastasis (pT1a pN0 pM0 pStage 0)

Endoscopy revealed a squamocolumnar junction 20 cm from the incisor teeth and a type 0-IIb + IIc tumor with indistinct boundaries 30 cm from the incisor teeth (Fig. 3). Mucosal biopsy revealed adenocarcinoma.

Computed tomography detected no enlarged lymph nodes and no other distant metastatic sites. Following diagnosis of cT1aN0M0 based on the Union for International Cancer Control TNM Classification 8th edition, subtotal esophagectomy was performed with lymph node and neck dissection. The postoperative pathological diagnosis was of well-differentiated adenocarcinoma (143 × 56 mm) with no lymph node metastasis (pT1a pN0 pM0 pStage 0 based on the Union for International Cancer Control TNM Classification 8th edition; Fig. 3). The patient was found to be alive and recurrence-free 3 months after the operation.

## Discussion

BE was named after a British thoracic surgeon, Norman Rupert Barrett (1903–1979), who reported a patient showing presence of columnar epithelium covering the lower esophagus with an esophageal ulcer [1].

BE indicates the origin of adenocarcinoma. Especially in the United States of America, histologically, esophageal adenocarcinoma has overtaken esophageal squamous cell carcinoma since 1995, and in 2005, up to 70% of esophageal cancers were BEAC [2].

Gastroesophageal reflux disease (GERD) is well recognized as a major cause of BE and BEAC. Among the reflux contents, gastric acid and bile acid reflux are of critical importance [3].

One systematic review uncovered that the prevalence of GERD was 18–27% in North America, 8–25% in Europe, 2–7% in East Asia, and 23% in South America [4]. The prevalence of GERD has been increasing, especially in developed countries, and the associated BEAC is increasing in North America, Europe, and Australia [5].

In Japan, BE is classified according to its length; when it is 3 cm or larger, it is called LSBE, and when it is shorter than 3 cm, it is called short segment BE (SSBE) [6].

The detection frequency of LSBE larger than 13 cm is 1.27% (15/1175) [7], and it is conceivable that LSBE larger than 15 cm is a relatively uncommon condition. Until now, nine cases have been reported in the literature [8–15] (Table 1).

**Table 1:** Characteristics of patients with LSBE larger than 15 cm

Case no.	Author	Year	Age	Gender	Length of Barrett's esophagus	Size of carcinoma (cm)	Location of the tumor	Histology	Depth of invasion	N	ly	V	Background disease	Prognosis
1	Kato	2002	45	M	15	2.7 × 2.3	Lt	tub2	SM	0	0	0	Hiatus hernia	2Y9M alive
2	Fujiwara	2006	67	F	18	5	UtMt	tub1	SM	0	2	0	Kyphosis, hiatus hernia	4Y8M alive
3	Fujiwara	2006	57	M	22	2	Lt	tub1	M	0	0	0	Post-total gastrectomy (R-Y, 29 years ago)	2Y alive
4	Stefan Hartl	2008	58	M	15	3	Lt	tub1	M	0	–	–	–	1M EMR, 4M additional operation
5	Shimada	2013	74	M	20	2.0 × 3.0	UtMt	tub1	M	0	–	–	Post-total gastrectomy (R-Y, 44 years ago)	1Y10M alive
6	Morita	2013	74	M	17	2.5 × 1.9	Ae	tub1	SM	0	0	0	Hiatus hernia	3Y alive
7	Kikuchi	2016	63	M	18	18 × 4	MtLt	tub1, pap > tub2, por	MP	0	0	0	Hiatus hernia	1Y6M alive
8	Zaiki	2016	65	M	15	12	LtMtAe	tub1	M	0	0	0	Hiatus hernia	1Y6M alive
9	Miyazaki	2016	68	M	17	11.6 × 4.9	LtMtAe	tub2 > tub1 > por	SM	0	1	0	Hiatus hernia	2Y5M died
10	Our case	2020	66	M	16	7 × 4.5	LtAe	por2 > sig	AD	N3	1	2	Hiatus hernia	2Y lymph node recurrence, alive
11	Our case	2020	83	F	15	6.0 × 5.2	Ut	tub1, tub1	AD	N3	2	2	Hiatus hernia	2M lymph node recurrence, died
12	Our case	2020	66	M	18	14.3 × 5.6	UtMtLtAeG	tub1	SM	0	0	0	Hiatus hernia	3M alive

*tub1* well-differentiated tubular adenocarcinoma, *tub2* moderately differentiated tubular adenocarcinoma, *pap* papillary adenocarcinoma, *por* poorly differentiated adenocarcinoma, *M* mucosal layer, *SM* submucosal layer, *MP* muscularis propria, *AD* adventitia, *EMR* endoscopic mucosal resection

In these cases, including ours, 10 patients were men, and the average age was 65.5 years. The median length of BE was 17 cm (15–22 cm). The depth of invasion was T1a, T1b, T2, and T3 in 4, 5, 1, and 2 cases, respectively. Two of the three cases we encountered had advanced cancer with early recurrence. In one case, the enlarged lesion occupied almost the entire BE. Overall, 4 of 12 cases had superficial lesions (cases 7, 8, 9, 12). In some cases, the advanced cancer is associated with superficial, enlarged lesions, and preoperative diagnosis of the resection area is important. In such cases, marginal resection is required, and total resection of BE may be preferable owing to the heterogeneity of BEAC derived from LSBE.

The treatment policy for BEAC depends on the depth of invasion and the stage of the disease as in the case of esophageal cancer. We performed surgery after three courses of SOX therapy in case 1. The effectiveness of preoperative chemotherapy for esophageal cancer has been reported, and the National Comprehensive Cancer Network guideline also recommends preoperative chemotherapy as an option for esophageal adenocarcinoma with lymph node metastases deeper than T2 [16, 17]. A clinical trial of preoperative SOX therapy for esophageal adenocarcinoma of Siewert type I or II with esophageal invasion larger than 3 cm with lymph node metastasis regardless of T3/T4a or T factor is ongoing in Japan.

Overall, long-term recurrence-free survival is achieved, except in patients with advanced cancer and in case 4, early curative resection may improve the prognosis of BE cancer. Based on these findings, residual BE surveillance and prevention of BEAC are critical.

Heartburn and esophageal reflux were observed in 8 of the 12 cases. Gashi et al. [18] reported that BE shortened the maximum length and total circumference when proton pump inhibitors (PPIs) were administered for 2 years. As such, PPIs may be effective in patients with reflux esophagitis and GERD.

There were two cases of BE after total gastrectomy. In an animal model, Miwa et al. [19] reported that the presence of bile acids and gastric juice was important for the development of BE and adenocarcinoma. It is suggested that bile acid reflux influences the occurrence of BEAC in two cases after total gastrectomy. Shirvani et al. [20] reported that stimulation of the BE mucosa with bile acid increased the expression of cyclooxygenase 2 (COX2); it is considered to suppress apoptosis via prostaglandin E2 by expression of COX2. COX2-selective inhibitors and aspirin may increase apoptosis, suppress growth of esophageal adenocarcinoma, and shorten BE [21–23].

LSBE surveillance is another important consideration for future research. Annual surveillance of all BE cases is financially inefficient, and it is important to identify BE with a high carcinogenic risk and apply an efficient method for narrowing the cases that require close surveillance.

In 2014, the British Gastroenterological Society recommended surveillance every 2–3 and 3–5 years for LSBE 3 cm or larger and SSBE shorter than 3 cm, respectively [24].

Rajeswari et al. reported that BE length may be a risk for BEAC. The annual risk of BEAC stratified by length is 0.31%/year (3 cm or shorter), 0.97%/year (4 to 6 cm), 1.26%/year (7 to 9 cm), 1.64%/year (10 to 12 cm), and 2.41%/year (13 cm or larger). Patients with carcinogenesis within a year have significantly longer BE length, and a 28% increase in annual risk of BEAC was seen for every 1 cm of BE length increase. Ultra-long BE is a risk factor for carcinogenesis, and for LSBE > 15 cm, short-term follow-up within at least 1 year is considered necessary.

Random biopsy is recommended for surveillance of BEAC in Europe and America; magnifying endoscopy is not recommended. For early lesions, the general strategy is to perform radiofrequency ablation (RFA) on the remaining BE after removal by endoscopic mucosal resection.

However, although RFA is an option for LSBE treatment and prevention of cancer development, it is not particularly common in Asian countries. Further reporting and accumulation of therapeutic results is necessary for its dissemination. The patient in case 6 underwent endoscopic submucosal dissection. Narrow-band imaging (NBI) allowed accurate recognition of the lesion position and curative resection, and 3-year recurrence-free survival was achieved. Sharma et al. and Curvers et al. [25, 26] reported that biopsy using NBI was useful; however, further work remains to be done as NBI was expected to be efficient for future surveillance.

## Conclusions

BEAC might show good prognosis if detected and treated early. Extremely LSBE is associated with a high incidence of BEAC; therefore, early detection and treatment with close surveillance is essential. Further case accumulation is warranted for prevention and establishment of surveillance.

## Data Availability

Data sharing is not applicable to this article, as no datasets were generated or analyzed during the study.

## Abbreviations

BE: Barrett's esophagus
BEAC: Barrett’s esophageal adenocarcinoma
COX2: Cyclooxygenase 2
ESD: Endoscopic submucosal dissection
GERD: Gastroesophageal reflux disease
LSBE: Long-segment Barrett's esophagus
NBI: Narrow-band imaging
PPI: Proton pump inhibitor
RFA: Radiofrequency ablation
